# Metrics of Patient, Public, Consumer, and Community Engagement in Healthcare Systems: How Should We Define Engagement, What Are We Measuring, and Does It Matter for Patient Care? Comment on "Metrics and Evaluation Tools for Patient Engagement in Healthcare Organization- and System-Level Decision-Making: A Systematic Review"

**DOI:** 10.15171/ijhpm.2018.94

**Published:** 2018-10-02

**Authors:** Zackary Berger

**Affiliations:** ^1^Division of General Internal Medicine, Johns Hopkins School of Medicine, Baltimore, MD, USA.; ^2^Johns Hopkins Berman Institute of Bioethics, Baltimore, MD, USA.

**Keywords:** Patients, Decision-Making, Engagement, Systems, Shared Decision-making, Systematic Reviews

## Abstract

In a rigorous systematic review, Dukhanin and colleagues categorize metrics and evaluative tools of the engagement of patient, public, consumer, and community in decision-making in healthcare institutions and systems. The review itself is ably done and the categorizations lead to a useful understanding of the necessary elements of engagement, and a suite of measures relevant to implementing engagement in systems. Nevertheless, the question remains whether the engagement of patient representatives in institutional or systemic deliberations will lead to improved clinical outcomes or increased engagement of individual patients themselves in care. Attention to the conceptual foundations of patient engagement would help make this systematic review relevant to the clinical care of patients.

While patient engagement is often mentioned as an important goal for healthcare organizations, how is it to be defined and measured, and what is its relevance for clinical care? In this rigorous and detailed systematic review by Dukhanin et al,^1^ the authors come to informative, helpful conclusions about the very nature of patient engagement and patient engagement metrics, even granting the limitations of their dataset and systematic reviews in general.

The first question concerns the meaning of engagement, or, rather, “patient, public, consumer and community (P2C2) engagement in organization-, community-, and system-level healthcare decision-making.” For the purposes of this review, the authors defined P2C2 engagement as “a continuous systematic effort to incorporate the needs, values, and preferences of the P2C2 engagement participants into decision-making.” That is to say, engagement involves participants as representatives of their communities or constituencies rather than focusing on the engagement of individuals in decision-making themselves. While the authors of any systematic review are free to define their topic as they wish for reasons of feasibility and clarity, the reader may fairly ask whether their definition serves a clinical purpose. Is it the case that including representatives of patient groups will lead to engagement of patients as individuals, or restructuring of healthcare systems to take patient concerns into account?

We will consider the potential clinical relevance of this review in our conclusion, but one alternative way to consider the conceptual framework of patient engagement at multiple levels, rather than solely the organization-, community- and systems-level, is as a multicomponent intervention, including other additional dimensions. On the one hand, social and political axes of engagement might enable greater exercise of power by community organizations and individual patients; and by redistributing resources to economically disadvantaged patients. Individual axes of engagement might involve the use of shared decision-making.^2^

What studies were considered in this review? The authors included studies from all countries, not just the United States, and studies from 1962 onward. In so doing, the authors recognized that patient engagement is not an innovation of late vintage, but the subject of repeated attempts and initiatives on the part of healthcare organizations and systems for decades. (Perhaps the fact that patient engagement has been a long-standing topic of research indicates the difficulties associated with effective implementation.)

Most full-text articles included in this current review identified tools implemented in the United States (12 of 23 tools), 3 in Canada, 3 in the United Kingdom, 2 in Nepal; the rest described tools implemented in assorted low- or middle-income (Djibouti, Honduras, South Africa, or Tanzania) or high-income countries (Ireland or New Zealand).

How was patient engagement assessed in the included studies? The authors first categorized included articles depending on whether they addressed outcome or process. Much as diabetes affects both measures of blood sugar and the clinical outcomes of interest, patient engagement can be assessed via the processes which reflect such engagement or the outcomes which are the result of such engagement. Process and outcome were further categorized by subdomain and metric within that subdomain; we consider these below.

Outcome domains were subcategorized into internal outcomes (relevant to and evaluated within a healthcare organization or system), external outcomes (eg, relating to population health or influence on the broader public) or cost-effectiveness, further categorized into “internal” and “external” aspects.

Process metrics were categorized into four subdomains: direct process metrics (evaluating the degree of direct control P2C2 have over the decision-making process); surrogate process metrics (which measure formal elements of the decision-making processes, not direct control, eg, whether patients have veto power); preconditions for engagement metrics (eg, characteristics which make it possible for there to be patient participation – for example, parking arrangements or compensation for participation); and, finally, aggregate process metrics, which assess various domains of the process and are meant to provide summary measures.

Thus, the included studies comprise a suite of measurement tools and metrics suitable for use in healthcare organizations. In addition, the organization of metrics according to the Arstein language of engagement will help practitioners prioritize different management strategies.

Two other findings are worth highlighting. First, some relevant domains are underemphasized or not addressed by the literature on patient engagement metrics. For example, only two tools measured improved trust in the organization, and no tool measured either sustainability of engagement or the capacity to increase or scale engagement. P2C2 participants’ involvement in finalizing decisions was evaluated in only one metric.

The authors limited their review to organization and system level decisions in which P2C2 could engage, as they say, “[Such domains] were excluded to focus our review, and because existing engagement frameworks consider engagement in organization-, community-, and system-level decisions as a conceptually distinct activity from engagement in more societal-level decisions.” The obvious question is whether a different scope of this research question might have led to different methods for assessing patient engagement. If patient advocacy were associated with greater influence in the design of national health systems, eg, in prioritizing biomedical approaches vs. social determinants of health, would that effect greater patient engagement? The fact that this would require a differently scoped systematic review does not mean that such a broader look is not potentially relevant.

To revisit our initial question, does engagement of P2C2 within a given healthcare organization achieve patient-important or clinically relevant outcomes (not to mention public health or population outcomes, which this review explicitly does not consider)? In other words, where should engagement take place, and where should patient (or consumer or public) influence act in order to be truly felt in healthcare? Perhaps disaggregating the P2C2 group would lead to answers in this regard.

Lastly, it is possible that some kinds of patient engagement were not captured in this review. A recent (2018) scoping review by Scholl et al^3^ considers institutional and organizational characteristics that influence the implementation of interventions to encourage shared decision-making in healthcare settings; one can argue that SDM is itself a means to patient engagement. The review addressed not metrics (as in this work) but characteristics of systems; thus perhaps the two bodies of evidence can be combined to provide a road map for engagement with patients in decision-making on an organizational level. This would also require a broadened understanding of SDM, which has generally been taken to apply to the individual encounter between doctor and patient. Whether SDM at the systems level means merely encouraging SDM at the patient-provider level, or if – in addition – this implies sharing of systems-level decisions with patients, is a question that does not yet have a clear answer.

Equipped with this review and detailed understanding of how exactly researchers, clinicians, and patients can go about measuring patient engagement, we can take further steps to actually improving its implementation in the world.

## Ethical issues

Not applicable.

## Competing interests

Author declares that he has no competing interests.

## Author’s contribution

ZB is the single author of the paper.
